# Identifying Predictors of Serious Adverse Events in Antidepressant Treatment from a Decade-Long Nationwide Pharmacovigilance Study: Impact of Dementia and Parkinson’s Disease Treatment

**DOI:** 10.3390/medicina61061103

**Published:** 2025-06-17

**Authors:** Jungmin Han, Minsung Kim, Yujin Kim, Soo Hyeon Lee, Sooyoung Shin, Yeo Jin Choi

**Affiliations:** 1 Department of Pharmacy, School of Pharmacy, Kyung Hee University, Seoul 02447, Republic of Korea; 2 Department of Pharmacy, School of Pharmacy, Sookmyung Women’s University, Seoul 02447, Republic of Korea; 3 Department of Regulatory Science, Graduate School, Kyung Hee University, Seoul 02447, Republic of Korea; 4 Institute of Regulatory Innovation through Science (IRIS), Kyung Hee University, Seoul 02447, Republic of Korea; 5 Department of Pharmacy, College of Pharmacy, Ajou University, Suwon 16499, Republic of Korea; 6 Research Institute of Pharmaceutical Science and Technology (RIPST), Ajou University, Suwon 16499, Republic of Korea

**Keywords:** adverse drug events, drug safety, depression, Parkinson’s disease, dementia, antidepressants

## Abstract

*Backgrounds and Objectives:* This study aims to characterize the prevalence and severity of antidepressant-associated adverse drug events (ADEs) and to identify predictors strongly associated with serious adverse events (SAEs). *Materials and Methods:* Disproportionality analysis on antidepressant-related ADEs spontaneously reported to the Korea Adverse event Reporting System (KIDS KAERS DB) from 2014 to 2023 was performed. Multiple logistic regression was conducted to identify predictors associated with SAEs. Sensitivity analysis was performed to validate the overall findings and assess the robustness of associations across subgroups defined by completeness of demographic data (age and sex), elderly age-stratification, and causality assessment. The study protocol was approved by the Kyung Hee University institutional review board. *Results:* Among 21,103 antidepressant-related ADEs, duloxetine was the most etiologic medication, followed by amitriptyline and escitalopram. Fluoxetine is the only agent with a high likelihood of reporting SAEs. ADEs involving vascular (extracardiac) disorders (ROR 42.42, 95% CI 13.19–136.42) and liver and biliary system disorders (ROR 7.84, 95% CI 3.77–16.29) were most likely to be SAEs. The predictors associated with substantial increased SAE risk were fluoxetine use (OR 2.71, 95% CI 1.68–4.39), male sex (OR 1.48, 95% CI 1.11–1.98), and concomitant administration of antiparkinsonian treatment (OR 8.29, 95% CI 3.61–19.06) and antidementia treatment (OR 2.94, 95% CI 1.34–6.05). Sensitivity analyses demonstrated similar and consistent findings. However, reversed trends in the association between SOC-based ADEs and sex were observed in the sensitivity analysis restricted to cases with “certain” and “probable” causality. *Conclusions:* The type of antidepressant, concomitant medications, and sex are major predictors for SAE risk. Further controlled studies on the impact of comorbidities and polypharmacy on antidepressant-related SAEs are warranted.

## 1. Introduction

Pharmacovigilance refers to the science and activities pertaining to the detection, assessment, and prevention of adverse drug events (ADEs) or medication-related problems [1]. The primary goal is to ensure and enhance patient and drug safety by detecting previously unknown ADEs and identifying risk factors for potential ADEs [1]. While the majority of ADEs are self-limiting, serious ADEs can lead to life-threatening reactions, long-term disability, and even death, potentially endangering patient safety [2]. In fact, ADEs have become a major contributor to global mortality, with the mortality rate rising from 2.05 to 6.86 per 100,000 population between 2001 and 2019 [1,3,4]. Notably, adult men aged 20–50 years have been reported to experience a disproportionately high ADE-related mortality rate [4]. The risk is even higher among elderly patients, who are more vulnerable due to the effects of age-related changes in pharmacokinetic and pharmacodynamic properties, the presence of multiple comorbidities, and polypharmacy [1,5,6,7].

Depression, also known as major depressive disorder (MDD), is one of the most prevalent psychiatric disorders worldwide, affecting approximately 350 million patients globally [8,9]. The World Health Organization (WHO) ranked MDD as the third leading cause for global disease burden, with predictions that it will become the leading cause by 2030, emphasizing the urgent need for optimal MDD treatment [10]. Antidepressants, including selective serotonin reuptake inhibitors (SSRIs) and serotonin–norepinephrine reuptake inhibitors (SNRIs), are the mainstay of MDD management, with growing evidence for individualized treatment options based on patient acceptability [11]. However, the overwhelming components of MDD treatment, which consequently hinder MDD treatment, is the complexity of symptoms that involve psychiatric or emotional, physical, and cognitive systems, alongside ADEs associated with antidepressants. These ADEs can negatively impact medication adherence and overall treatment outcomes [12].

As the global prevalence of MDD continues to grow each year, antidepressant prescriptions have also increased. A recent study reported a 66.3% increase in antidepressant use from January 2016 to December 2022, especially after the COVID-19 pandemic, with a notable increase among young adults [13]. The substantial growth in antidepressant use has doubtlessly contributed to a marked increase in ADE cases related to MDD treatment [14]. Previous studies have identified various antidepressant-associated ADEs, including an increased risk of falls and comorbid psychiatric conditions such as attention deficit/hyperactivity disorder (ADHD) [15,16]. Furthermore, MDD patients are at higher risk of developing other psychiatric disorders, particularly anxiety, which may predispose MDD patients to elevated ADE risks associated with multiple comorbidities and concomitant medications [17]. Despite these concerns, a comprehensive pharmacovigilance investigation utilizing real-world data (RWD) on MDD treatment is currently lacking [18]. Moreover, investigations on the predictors of serious adverse events (SAEs) across various antidepressant classes and concomitant medications are still limited, despite the substantially high SAE incidence from antidepressant use. Hence, this study aims to comprehensively characterize the prevalence and severity of ADEs related to antidepressant treatment and to identify predictors strongly associated with SAEs by utilizing a spontaneous adverse event reporting system to promote safe medication use in patients.

## 2. Materials and Methods

### 2.1. Study Design and Data Collection

This cross-sectional study was conducted in accordance with Strengthening the Reporting of Observational Studies in Epidemiology (STROBE) guidelines [19]. This study analyzed adverse event (AE) cases related to antidepressant treatment that were spontaneously reported to the Korean Adverse Event Reporting System database (KIDS KAERS DB) from 1 January 2014 to 31 December 2023. The KIDS KAERS DB was constructed by the Korean Institute of Drug Safety and Risk Management (KIDS, Ministry of Food and Drug Safety) [20]. The prespecified medication classes for depression treatment included SSRIs, SNRIs, tricyclic antidepressants (TCAs), and other depression treatments. A total of 15 medications treated for depression were included: amitriptyline, bupropion, citalopram, clomipramine, duloxetine, escitalopram, fluoxetine, imipramine, mirtazapine, paroxetine, sertraline, tianeptine, trazodone, venlafaxine, and vortioxetine. All antidepressant-related AEs with a causality assessment of “certain”, “probable/likely”, and “possible” according to World Health Organization-Uppsala Monitoring Centre (WHO-UMC) criteria were included and analyzed [21]. The prespecified exclusion criteria were as follows: irrelevant ADE cases that were classified as “unlikely”, “conditional/unclassified”, or “unassessable/unclassifiable” according to WHO-UMC criteria, and those with masked (MSK-coded) etiologic medications. The KIDS KAERS DB assigns an MSK code to medication products that are marketed by fewer than 2 pharmaceutical companies. The severity of ADE reactions was reported to the KIDS KAERS DB in accordance with the International Conference on Harmonization (ICH) E2D guideline and were further classified into system organ classes (SOCs) [22]. The ICH E2D guideline classifies an SAE as any ADE involving congenital abnormalities or birth defects, hospitalization or prolonged existing hospitalization, persistent or significant disability or incapacity, life-threatening conditions, death, or other medically significant events [22]. The following data were extracted from the KIDS KAERS DB: (1) patient demographic information (sex and age), (2) ADE information (including etiologic medication, occurrence date, causality assessment, and seriousness), and (3) medical histories and concurrent medications. The protocol for utilizing the KIDS KAERS DB was approved by the KIDS (Ministry of Food and Drug Safety) (KIDS KAERS DB 2405A0009) and the Kyung Hee University institutional review board (IRB) (No. KHSIRB-24-417 (EA), approved 14 August 2024).

### 2.2. Statistical Analysis

Descriptive statistics were performed to summarize patient demographics and ADE types related to depression treatment. Age was expressed as the mean ± standard deviation based on the Kolmogorov–Smirnov normality test. A disproportionality test was performed to determine the likelihood of reporting SAEs for ADEs with at least 4 reported cases of both nonserious ADEs and SAEs to ensure the validity and reliability of the results [2,23]. The disproportionality test was conducted for the following assessments based on READUS-PV reporting guidelines [24]: (1) the association between antidepressant agents and SAEs, (2) the association of SOC-based ADEs with seriousness, and (3) the association of the SOC-based ADEs with sex. The effect size of the disproportionality analysis was estimated as reporting odds ratios (RORs) with corresponding 95% confidence intervals (CIs), with Mantel–Haenszel adjusted *p*-values. A univariate analysis was performed to identify predictors associated with the seriousness of ADEs related to depression treatment, including the factors of patient sex, age, number of concomitant drugs, types of concomitant medications, and medication types for depression treatment. Multiple logistic regression with the forward selection method was conducted to estimate the effect size of predictors that were significantly associated with SAEs based on the univariate analysis. The effect size was estimated as the odds ratio (OR) with 95% CIs. Sensitivity analysis was designed to assess the robustness of the observed associations and minimize potential bias arising from missing information, aging-related factors, and limitations in causality analysis. Three sensitivity analyses were performed: (1) restricting the dataset to cases with a causality assessment of “certain” or “probable” to strengthen the validity of the causality assessment, (2) conducting age-stratification, focusing on patients aged 60 years and older to explore age-related patterns of SAEs, particularly among those treated with anti-dementia or anti-Parkinson’s disease medications, and (3) analyzing only cases with complete data on both age and sex to reduce the impact of incomplete demographic information. All statistical analysis was conducted with SPSS Statistic 26.0 (IBM SPSS Statistics for Windows, Armonk, NY, USA), and any *p*-value < 0.05 was considered statistically significant.

## 3. Results

### 3.1. Baseline Characteristics

Among 7,134,485 ADE cases extracted from the KIDS KAERS DB, a total of 21,103 ADE cases related to depression treatment from 1 January 2014 to 31 December 2023 were included in the analysis. The highest number of ADEs were reported in patients aged between 60 and 69 (n = 3544, 16.79%), followed by those between 70 and 79 (n = 3013, 14.28%), and approximately 65.70% of ADEs were reported in women (Table 1).

**Figure 1:**
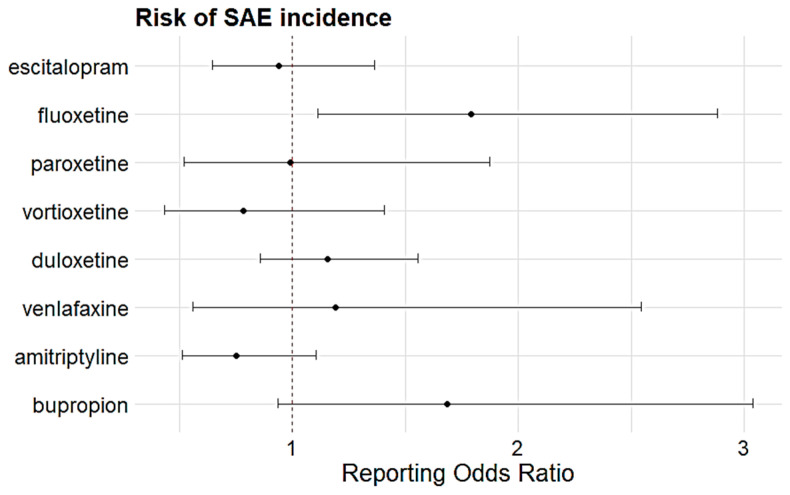
Disproportionality analysis on association between reporting of SAEs and etiologic agents. Black dots represent RORs and the black lines indicate 95% CI.

The prevalence of serious adverse events (SAEs) was 0.95% (n = 201). The most common etiologic medication class was SSRIs (n = 8247, 39.08%), followed by SNRIs (n = 6801, 32.22%). Duloxetine was the most common etiologic antidepressant (n = 6180, 29.28%), followed by amitriptyline (n = 4111, 19.48%) and escitalopram (n = 3649, 17.29%) (Table 2). Only fluoxetine had a significantly elevated likelihood of reported SAEs (ROR 1.79, 95% CI 1.11–2.88) (Figure 1).

### 3.2. ADE Types and Risk of Reporting SAEs

**Figure 2:**
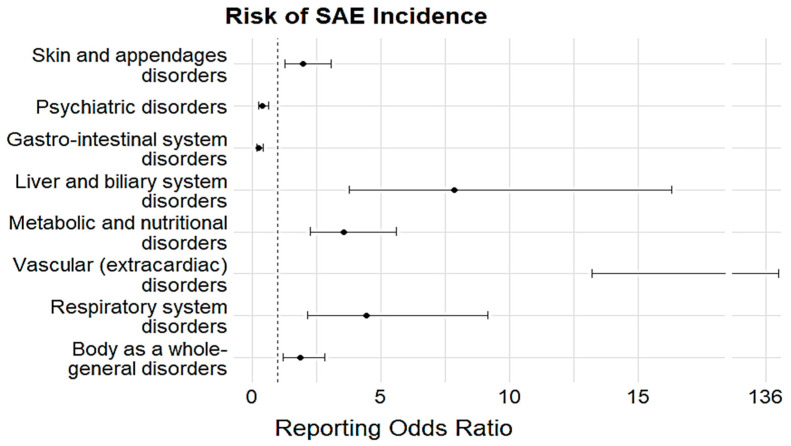
Disproportionality analysis of the association between SOC-based ADEs and seriousness. Black dots represent RORs and the black lines indicate 95% CI.

The most frequent types of depression treatment-related ADEs were associated with gastrointestinal disorders (n = 6354, 30.11%), followed by psychiatric disorders (n = 4657, 22.07%) and central and peripheral nervous system disorders (n = 4302, 20.39%) (Table 3). The etiologic medications with the highest ADE reports pertaining to psychiatric disorders were escitalopram (n = 932, 25.54%), fluoxetine (n = 347, 29.63%), amitriptyline (n = 1077, 26.20%), bupropion (n = 200, 25.97%), and mirtazapine (n = 78, 31.71%). The highest number of ADE cases related to gastrointestinal disorders was reported with paroxetine (n = 295, 27.83%), sertraline (n = 212, 26.90%), vortioxetine (n = 593, 37.58%), duloxetine (n = 2438, 39.45%), venlafaxine (n = 190, 30.60%), imipramine (n = 166, 21.56%), and trazodone (n = 123, 25.20%). ADEs associated with vascular (extracardiac) disorders were associated with the highest likelihood of being reported as SAEs (ROR 42.42, 95% CI 13.19–136.42, *p* < 0.05), followed by liver and biliary system disorders (ROR 7.84, 95% CI 3.77–16.29, *p* < 0.05) and respiratory system disorders (ROR 4.45, 95% CI 2.16–9.15, *p* < 0.05) (Figure 2). Other SOCs involving skin and appendage disorders (ROR 1.96, 95% CI 1.26–3.07, *p* < 0.05), metabolic and nutritional disorders (ROR 3.55, 95% CI 2.24–5.61, *p* < 0.05), and body-as-a-whole general disorders (ROR 1.89, 95% CI 1.22–2.84, *p* < 0.05) were also associated with a significantly higher likelihood of being reported as serious ADEs related to depression treatment (Figure 2).

**Figure 3:**
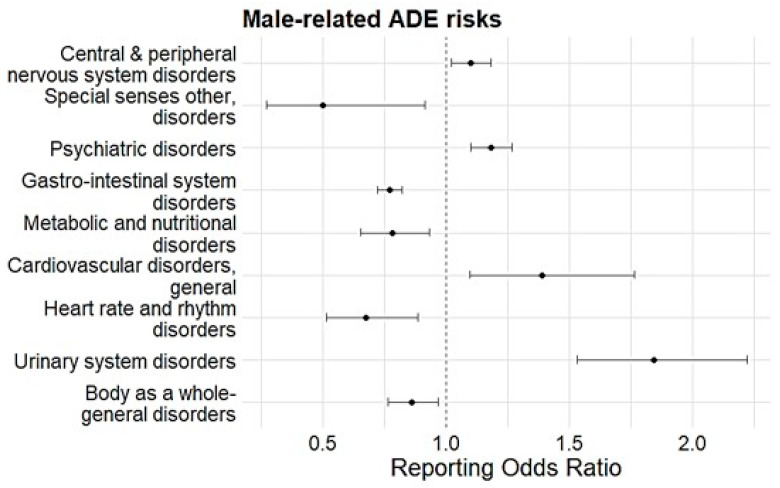
Disproportionality analysis on the association of SOC-based ADEs with sex. Black dots represent RORs and the black lines indicate 95% CI.

Men had a higher reporting risk of central and peripheral nervous system disorders (ROR 1.10, 95% CI 1.02–1.18, *p* < 0.05), psychiatric disorders (ROR 1.18, 95% CI 1.10–1.27, *p* < 0.05), general cardiovascular disease (ROR 1.39, 95% CI 1.10–1.77, *p* < 0.05), and urinary system disorders (ROR 1.84, 95% CI 1.53–2.22, *p* < 0.05) (Figure 2). On the other hand, men had a lower reporting risk of ADEs related to special sense disorders (ROR 0.50, 95% CI 0.27–0.92, *p* < 0.05), gastrointestinal disorders (ROR 0.77, 95% CI 0.72–0.82, *p* < 0.05), metabolic and nutritional disorders (ROR 0.78, 95% CI 0.66–0.93, *p* < 0.05), heart rate and rhythm disorders (ROR 0.68, 95% CI 0.52–0.89, *p* < 0.05), and body-as-a-whole general disorders (ROR 0.86, 95% CI 0.76–0.97, *p* < 0.05) (Figure 3).

### 3.3. Identification of Predictors

**Figure 4:**
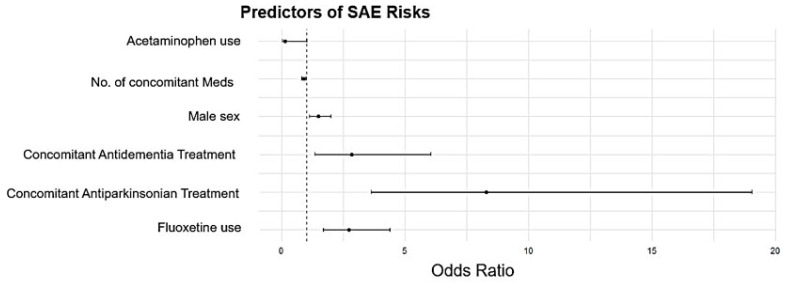
Predictors of antidepressant-associated SAE risk. Black dots represent RORs and the black lines indicate 95% CI.

The univariate analysis identified patient sex, number and type of concomitantly administered medications, and types of depression treatment as predictors associated with SAEs (Figure 4). Multivariate analysis demonstrated a substantially increased SAE risk with fluoxetine use (OR 2.71, 95% CI 1.68–4.39, *p* < 0.05), male sex (OR 1.48, 95% CI 1.11–1.98, *p* < 0.05), and concomitant administration of antiparkinsonian treatment (OR 8.29, 95% CI 3.61–19.06, *p* < 0.05) and antidementia treatment (OR 2.94, 95% CI 1.34–6.05, *p* < 0.05). Meanwhile, the risk of SAEs was significantly lower with an increasing number of concomitant medications (OR 0.90, 95% CI 0.82–0.98, *p* < 0.05) and concomitant acetaminophen use (OR 0.14, 95% CI 0.02–1.01, *p* < 0.05).

### 3.4. Sensitivity Analysis

The result of the sensitivity analysis on the association between antidepressant medication and reported SAEs is summarized in Table 4. Fluoxetine was associated with a substantially higher likelihood of reported SAEs (OR 2.62, 95% CI 1.58–4.36, *p* < 0.001) in ADE cases that included both age and sex information. Bupropion was associated with a substantially higher likelihood of reported SAEs across all sensitivity analysis. A substantially lower likelihood of reported SAEs was observed with amitriptyline in patients aged 60 years and older (OR 0.51, 95% CI 0.28–0.92, *p* < 0.05). Similar trends in the association between SOC and SAEs were observed across all sentivity analysis (Table 5). The likelihood of reporting an SAE was significantly higher for skin and appendages disorders, liver and biliary system disorders, metabolic disorders, respiratory system disorders, and body-as-a-whole general disorders. Notably, central and peripheral nervous system disorders were observed to have a higher likelihood of being reported as SAEs in sensitivity analyses.

The trends in the association between SOC-based ADEs and sex varied across the sensitivity analyses (Table 6). ADEs involved with psychiatric disorders and urinary system disorders were consistently associated with a high likelihood of SAE reporting. However, in the causality-restricted sensitivity analysis, these SOCs demonstrated inverse associations (psychiatric disorders: ROR 0.82, 95% CI 0.70–0.97, *p* < 0.05; urinary system disorders: ROR 0.49, 95% CI 0.31–0.76, *p* < 0.05). Similarly, gastrointestinal and general disorders showed increased SAE risk when causality was restricted to “certain” and “probable”, whereas these categories were associated with a decreased risk in the age- and sex- adjusted and age ≥ 60 years sensitivity analyses. ADEs involving skin and appendage disorders (ROR 1.24, 95% CI 1.01–1.53, *p* < 0.001), metabolic and nutritional disorders (ROR 8.53, 95% CI 4.93–14.75, *p* < 0.001), and platelet, bleeding, and clotting disorders (ROR 2.91, 95% CI 1.27–6.65, *p* < 0.05) had markedly elevated SAE risk in patients aged 60 years and older.

Similarly, the sensitivity analyses identified that concomitant use of antiparkinsonian and dementia treatment and fluoxetine use were major predictors associated with increased SAE risk (Table 7). Interestingly, SAE risk was elevated with increasing numbers of concomitant medications when ADE cases were restricted to “certain” and “probable” (OR 1.12, 95% CI 1.01–1.25, *p* < 0.05). Notably, aging was a strong contributor for SAE risk in elderly patients aged 60 years and older (OR 11.16, 95% CI 4.28–29.09, *p* < 0.001).

## 4. Discussion

This study comprehensively evaluated MDD treatment-related ADE records that were spontaneously reported to a nationwide pharmacovigilance system from 1 January 2014 to 31 December 2023. This study demonstrated that antidepressant-related ADEs were more commonly reported in women, and that the most common etiologic medication class was SSRIs, followed by SNRIs, with duloxetine being the most frequently used etiologic antidepressant. SAEs are more likely to be reported with fluoxetine. The most frequent ADE types were associated with gastrointestinal, psychiatric and central and peripheral nervous system disorders, with vascular (extracardiac) disorders being the MDD treatment agent with the highest risk of reported SAEs. The predictors associated with a substantial increased risk of overall SAEs were fluoxetine use, male sex, and concomitant administration of antiparkinsonian treatment and antidementia treatment.

This study indeed has several distinctive findings: Generally, polypharmacy or administration of multiple concomitant medications is often associated with an increased ADE risk due to potential drug–drug interactions [25,26]. However, in this study, the increasing number of concomitant medications was associated with decreased SAE risk from MDD treatment, which may seem counterintuitive. This finding may reflect the clinical behavior that healthcare professionals tend to take extra caution when patients receive multiple medications, potentially leading to the early detection and prevention of SAEs [27]. Considering that 36.34% of all ADE cases included in the study were from the elderly aged 60 years and older, and that age-stratified sensitivity analyses did not identify the number of concomitant medications as a critical predictor of increased SAEs, it is plausible that the observed phenomenon may be partially attributed to this intensified clinical monitoring and individualized medication management [28].

The current guidelines for MDD management highly recommend a more conservative approach when treating MDD in elderly patients, which involves lower starting doses, gradual dose titration, the selection of antidepressants with a low risk of drug–drug interactions, and cautious ADE monitoring [28]. These cautious prescribing practices may also explain the lower likelihood of reported SAEs associated with amitriptyline in patients aged 60 years and older in our sensitivity analysis, despite its classification as a high-risk anticholinergic agent per Beer’s criteria [29]. Medications such as certain antihypertensive agents, benzodiazepines, and corticosteroids may exacerbate or induce depression in the elderly, and healthcare professionals are often vigilant in identifying and managing these risks in the elderly [28]. Furthermore, stringent selection of antidepressants with favorable ADE profiles and appropriate monitoring may also have contributed to decreased antidepressant-related SAE risk with increased numbers of concomitant medications, reflecting endeavors to prevent drug–drug interactions associated with polypharmacy, as the majority of MDD treatment agents are known to have numerous drug–drug interactions.

However, it is important to note that this trend was reversed in the sensitivity analysis performed on ADE cases with a causality assessment of “certain” or “probable”. In this sensitivity analysis, a higher number of concomitantly administered medications was significantly associated with increased SAEs, implying that polypharmacy may indeed elevate this risk. This finding highlights the importance of accounting for potential reporting or attribution bias in spontaneous reporting systems [20,30]. Under-reporting in clinically complex patients, selective attribution of events to a primary drug rather than an interacting or concomitant agent, or differential thresholds for reporting based on case severity may all contribute to the observed discrepancies across analyses [30]. Hence, future controlled studies investigating antidepressant safety in relation to polypharmacy are warranted to evaluate the clinical impact of polypharmacy in depression patients and enhance patient prognosis in an evidence-based manner.

This study revealed an elevated likelihood of reported SAEs with fluoxetine use, and fluoxetine use itself may play as predictor for an increased risk of SAEs. One potential reason for this elevated SAE risk with fluoxetine lies with its unique pharmacokinetic properties [31]. Fluoxetine has a long half-life and undergoes extensive metabolic conversion to produce not only an active metabolite called norfluoxetine, but also several other metabolites [31]. Fluoxetine is a racemic mixture containing two enantiomers with variable potencies, and previous clinical studies have failed to determine the evident relationship between clinical outcomes and the plasma concentrations of either fluoxetine or norfluoxetine [31]. Evidence suggests that numerous cytochrome P450 (CYP) enzymes are involved with fluoxetine metabolism: CYP2D6, CYP2C19, CYP2C9, CYP3A4, and CYP3A5. Moreover, fluoxetine acts as a potent inhibitor against CYP2C19, CYP2C9, and CYP3A4, increasing the risk of clinically significant drug–drug interactions, especially in patients on multiple concomitant medications. These pharmacokinetic (PK) profiles may potentially increase the risk of adverse outcomes, especially in elderly patients or those with hepatic impairment [31,32]. Moreover, a recent study demonstrated that both fluoxetine and norfluoxetine increases the risk of cardiovascular and cerebrovascular diseases in geriatric patients [32]. Hence, fluoxetine use in older adults or those with comorbidities warrants cautious evaluation, and alternative antidepressants with more favorable safety profiles should be considered when appropriate.

The sensitivity analyses demonstrated a substantially high likelihood of reported SAEs with bupropion. Bupropion is a norepinephrine–dopamine reuptake inhibitor (NDRI) that provides a distinct pharmacological effect compared to other commonly used antidepressants. Bupropion has a favorable adverse event profile in terms of sexual dysfunction and weight gain; however, studies have revealed an increased risk of seizures, particularly at higher doses or in patients with predisposing factors [33]. Bupropion also has stimulating effects on the central nervous system, which may also exacerbate anxiety, insomnia, or agitation. Furthermore, bupropion is metabolized by CYP2B6 and is prescribed to depression patients who have other psychiatric comorbidities such as attention deficit hyperactivity or substance use disorders, which may contribute to a higher baseline risk of adverse events [33]. However, due to lack of comorbidity data, this study was not able to fully account for these potential confounders. Thus, further research incorporating comprehensive clinical information is required to identify high-risk subgroups and to provide safe and personalized medicine.

Another interesting finding was that concomitant administration of antiparkinsonian treatment and antidementia treatment substantially increased the likelihood of SAE development. Patients with neurological disorders such as Parkinson’s disease and dementia are at elevated risk of depression, and studies suggest that depression is often underdiagnosed and undertreated in patients with neurological disorders, despite the high prevalence of at least 40 to 50% [34,35]. Moreover, considering that patients diagnosed with PD and dementia often require multiple medication therapies, these patients are more susceptible to having complex medication regimens and an elevated risk of SAEs from drug–drug interactions, as well as comorbidities [36]. Neurological disorders including PD and dementia may alter PK and pharmacodynamic (PD) changes, and considering that these are age-related degenerative disorders and that age is also a major contributor to PK/PD alterations, these patients are particularly vulnerable to SAEs from antidepressant treatments [37]. Additionally, antidepressants, particularly medications with anticholinergic properties, may further increase the risk of falls, cognitive impairment, and cardiovascular events in elderly persons with neurodegenerative disorders, and the guidelines strongly recommend prescribing antidepressants with the lowest potential for anticholinergic effects in dementia patients [28].

Additionally, depression in patients with PD or dementia has a significant impact on patient prognosis and mortality [38]. One of the most common feature or signs of MDD in patients with neurological disorders is suicidal ideation, and a substantially higher suicide rate has been observed in Alzheimer’s and Parkinson’s disease patients [39]. Moreover, the risk of Alzheimer’s disease is substantially higher in depressive patients with mild cognitive impairment, implying faster cognitive decline and disease progression [38]. Nonetheless, the evidence on optimal MDD treatment in these patients is limited, and this study accentuates the crucial need for vigilant pharmacovigilance and cautious risk versus benefit assessment when prescribing antidepressants to patients with neurological diseases, particularly PD or dementia. Furthermore, given the substantial risks associated with polypharmacy and drug–drug interactions in these populations, the endorsement of multidisciplinary patient care is highly recommended, and further research on optimal MDD treatment in patients with neurological disorders is warranted to enhance patient safety. Meanwhile, clinicians are advised to implement structured medication reviews, monitor for potential drug–drug interactions, and tailor antidepressant selection, especially in older patients or patients with neurological comorbidities. Furthermore, routine monitoring of cognitive status, cardiac function, and metabolic panels are required to promote treatment safety and improve patient outcomes.

Male sex was identified as a significant risk factor for antidepressant-associated SAEs. Based on previous pharmacovigilance studies, men are at elevated risk of developing medication-associated SAEs than women, despite a lower overall rate of ADE reporting [2,40]. Although an evident mechanism or evidence on current findings is not available, several biological and behavior mechanisms may contribute to this disparity. From a pharmacological perspective, sex-related differences in PK/PD—particularly CYP450 enzyme activity—may influence drug metabolism and systemic exposure, potentially increasing vulnerability to adverse effects in men [41,42]. Moreover, emerging evidence indicates sex differences in depression-related pathophysiology, including anatomical brain abnormalities, impaired neuroplasticity, distinct transcriptional signatures in key brain regions, and differential immune activation patterns [43]. These biological differences may modulate both therapeutic potential and susceptibility to antidepressant-induced adverse outcomes.

This study revealed that men have a higher likelihood of reporting SAEs resulting in central and peripheral nervous system disorders, psychiatric disorders, general cardiovascular disorders, and urinary system disorders than women. SAEs from central and peripheral nervous system disorders and psychiatric disorders may be attributed to sex differences in neurotransmitter regulation in response to antidepressants or variation in healthcare utilization [44,45]. Men also have higher baseline cardiovascular risks such as for hypertension, coronary artery disease, and heart failure than women, which may have contributed to the higher number of SAE reports related to general cardiovascular disorders [46]. However, cautious interpretation of the results is necessary because of potential disparities in healthcare utilization between men and women [47]. Men are less likely to seek medical care than women unless the symptoms are severe or substantially impacting their daily lives. This healthcare-seeking behavior may result to an under-reporting of mild to moderate ADEs in men [47]. Moreover, studies have demonstrated sex disparities across ADE types, often showing a female predominance in overall ADE reporting [47]. This is supported by our sensitivity analyses restricted to cases with a causality assessment of “certain” or “probable”, which revealed an inversed pattern for SOC-based ADEs in men. Causality determination of an ADE often relies on limited clinical information and is subject to heterogeneity in reporter judgement, which can introduce classification bias. This variability may also lead to selection bias, as cases deemed more clinically evident or severe are more likely to be classified as having a “certain” or “probable” causal relationship. This represents a key limitation of the current study, and further studies incorporating standardized causality assessment methods and sufficient clinical data are warranted to validate the observed associations, reduce misclassification, and improve the reliability of sex-stratified safety assessments. Nonetheless, these findings highly indicate the importance of considering sex-based differences in pharmacovigilance activities, including medication use and monitoring. Hence, healthcare professionals should assess sex-related cardiovascular, neurological, and psychiatric comorbidities when initiating antidepressant therapy, and cautious and routine monitoring are highly warranted to optimize treatment safety.

This is the first nationwide pharmacovigilance study that has identified predictors associated with antidepressant-induced SAEs using RWD over 10-year period. The greatest benefit of RWD-based pharmacovigilance investigation is the ability to create evidence of drug safety derived from actual clinical practices, offering insights that may be more generalizable than those from clinical trials. However, there are several limitations should be acknowledged: First, the KIDS KAERS DB is a spontaneous, voluntary reporting database, which is inherently subject to under-reporting and reporting bias, where only a subset of actual adverse events is captured. This may result in the over-representation of severe cases, while underestimating mild or moderate adverse events, potentially affecting the validity and generalizability of our findings. Second, caution should be exercised when interpreting the study results due to potential issues with data completeness and quality. As discussed previously, missing information on comorbidities (cardiovascular, neurological, hepatic, or renal conditions) and the severity of depression may have influenced the observed associations and limited the ability to fully understand the scope of the patients involved. These unmeasured confounders could lead to the underestimation or misinterpretation of the risk factors contributing to antidepressant-induced SAEs and limit the ability to interpret causality. Future studies incorporating detailed clinical data, including psychiatric and medical histories, are warranted to accurately assess risk factors and strengthen the validity of causal inference. Third, the cross-sectional nature of the data precludes any determination of temporal or causal relationships between predictors and SAEs. Hence, further longitudinal or controlled studies are warranted to validate these findings to determine the causality and impact of these predictors. Additionally, as the data were collected exclusively from Korea, regional factors such as genetic predispositions, healthcare systems, and prescribing patterns limit the generalizability of the results to other ethnic populations. Thus, contextual factors should be considered when applying our findings to different healthcare settings or populations. Fourth, variability in sex-stratified sensitivity analyses of restricting cases to “certain” or “probable” causality suggest potential sex-related disparities in reporting or classification that may limit the interpretation of sex-based risk estimates. Further research using sex-disaggregated clinical data and a standardized causal attribution method is needed to clarify these differences. Despite these limitations, the Korea Institute of Drug Safety and Risk Management (Ministry of Food and Drug Safety), which performs rigorous in-depth investigations of reported ADEs through chart reviews and expert consultations, maintains data reliability, thereby strengthening the validity of the findings. Moreover, this study conducted sensitivity analysis to minimize the potential bias arising from missing information and limitations in causal inference. This study contributes to the growing body of literature guiding safer antidepressant use and accentuating the importance ongoing pharmacovigilance efforts. This study also emphasizes the need for continued research to raise the awareness of antidepressant-induced ADEs. Furthermore, this study offers tailored guidance on SAE prevention that can be integrated into clinical practice, ultimately enhancing patient safety and treatment outcomes.

## 5. Conclusions

This study comprehensively evaluated antidepressant-associated ADE records that were spontaneously reported to a nationwide pharmacovigilance system from 1 January 2014 to 31 December 2023. This study suggests that the types of antidepressants and concomitantly administered medications prescribed along with patient sex are major predictors for SAE incidence. These findings emphasize the need for healthcare professionals to carefully assess the risk vs. benefit profile of depression treatment, particularly in patients with multiple comorbidities or taking multiple medications, and even more particularly in those with PD or dementia. Nevertheless, further studies controlling for the impact of comorbidities, polypharmacy, and individualized treatment regimens on antidepressant-related ADEs are required to develop more tailored and effective therapeutic strategies for MDD patients at higher SAE risk.

## Figures and Tables

**Table 1:** Demographic information.

Characteristics	No. of Cases (n)	Percentage
Sex ^a^		
Men	6305	29.88%
Women	13,862	65.70%
Age (56.5 ± 18.1) ^b^		
0~9	110	0.52%
10~19	371	1.78%
20~29	1177	5.58%
30~39	1213	5.75%
40~49	1787	8.47%
50~59	2917	13.82%
60~69	3544	16.79%
70~79	3013	14.28%
80~89	1061	5.03%
90~99	50	0.24%
Causality		
Certain	292	1.38%
Probable/Likely	3605	17.08%
Possible	17,206	81.53%
Seriousness		
Non-serious ADE	20,902	99.05%
Serious ADE	201	0.95%
Reporter Types		
Doctors	4230	20.04%
Pharmacists	8764	41.53%
Other Healthcare Professionals	5399	25.58%
General Public	2057	9.75%
Unknown	651	3.08%
Number of Concomitant Medications		
1	11,427	54.15%
2	2903	13.76%
3	2168	10.27%
4	1594	7.55%
≥5	3011	14.27%

^a^ missing in 936 cases (4.44%); ^b^ missing in 5860 cases (27.77%).

**Table 2:** Number of reported ADEs per medication.

	SAE (n = 201)	Non-SAE (n = 20,902)	Total (n = 21,103)
SSRI	77 (38.31%)	8170 (39.09%)	8247 (39.08%)
citalopram	0 (0.00%)	1 (0.00%)	1 (0.00%)
escitalopram	33 (16.42%)	3616 (17.30%)	3649 (17.29%)
fluoxetine	19 (9.45%)	1152 (5.51%)	1171 (5.55%)
paroxetine	10 (4.98%)	1050 (5.02%)	1060 (5.02%)
sertraline	3 (1.49)	785 (3.76%)	788 (3.73%)
vortioxetine	12 (5.97%)	1566 (7.49%)	1578 (7.48%)
SNRI	72 (35.82%)	6729 (32.19%)	6801 (32.22%)
duloxetine	65 (32.34%)	6115 (29.26%)	6180 (29.28%)
venlafaxine	7 (3.48%)	614 (2.94%)	621 (2.94%)
TCA	33 (16.42%)	4443 (21.26%)	4476 (21.21%)
amitriptyline	31 (0.1%)	4080 (19.52%)	4111 (19.48%)
clomipramine	0 (0.00%)	23 (0.11%)	23 (0.11%)
imipramine	2 (1.00%)	340 (1.63%)	342 (1.62%)
Others	19 (9.45%)	1560 (7.46%)	1579 (7.48%)
bupropion	12 (5.97%)	758 (3.63%)	770 (3.65%)
mirtazapine	3 (1.49%)	243 (1.16%)	246 (1.17%)
tianeptine	2 (1.00%)	73 (0.35%)	75 (0.36%)
trazodone	2 (1.00%)	486 (2.33%)	488 (2.31%)

Abbreviation: SAEs: serious adverse events; SNRI: serotonin and norepinephrine reuptake inhibitor; SSRI: selective serotonin reuptake inhibitor; TCA: tricyclic antidepressants. The grey shading indicates the classes of antidepressants.

**Table 3:** System Organ Class (SOC)-based ADEs classified by antidepressants.

	SSRI						SNRI		TCA			Others			
	Citalopram (n = 1)	Escitalopram (n = 3649)	Fluoxetine (n = 1171)	Paroxetine (n = 1060)	Sertraline (n = 788)	Vortioxetine (n = 1578)	Duloxetine (n = 6180)	Venlafaxine (n = 621)	Amitriptyline (n = 4111)	Clomipramine (n = 23)	Imipramine (n = 342)	Bupropion (n = 770)	Mirtazapine (n = 246)	Tianeptine (n = 75)	Trazodone (n = 488)
Skin and appendage disorders	0 (0.00%)	249 (6.82%)	92 (7.86%)	47 (4.43%)	40 (5.08%)	123 (7.79%)	350 (5.66%)	31 (4.99%)	216 (5.25%)	1 (4.35%)	14 (4.09%)	60 (7.79%)	13 (5.28%)	2 (2.67%)	15 (3.07%)
Musculo-skeletal system disorders	0 (0.00%)	46 (1.26%)	41 (3.50%)	7 (0.66%)	4 (0.51%)	8 (0.51%)	59 (0.95%)	10 (1.61%)	28 (0.68%)	0 (0.00%)	1 (0.29%)	22 (2.86%)	2 (0.81%)	1 (1.33%)	8 (1.64%)
Collagen disorders	0 (0.00%)	0 (0.00%)	0 (0.00%)	0 (0.00%)	0 (0.00%)	0 (0.00%)	1 (0.02%)	0 (0.00%)	0 (0.00%)	0 (0.00%)	0 (0.00%)	0 (0.00%)	0 (0.00%)	0 (0.00%)	0 (0.00%)
Central and peripheral nervous system disorders	0 (0.00%)	739 (20.25%)	193 (16.48%)	202 (19.06%)	153 (19.42%)	284 (18.00%)	1370 (22.17%)	118 (19.00%)	849 (20.65%)	0 (0.00%)	54 (15.79%)	175 (22.73%)	46 (18.70%)	16 (21.33%)	103 (21.11%)
Vision disorders	0 (0.00%)	30 (0.82%)	21 (1.79%)	17 (1.60%)	15 (1.90%)	18 (1.14%)	46 (0.74%)	7 (1.13%)	46 (1.12%)	0 (0.00%)	4 (1.17%)	7 (0.91%)	1 (0.41%)	1 (1.33%)	3 (0.61%)
Hearing and vestibular disorders	0 (0.00%)	10 (0.27%)	2 (0.17%)	2 (0.19%)	1 (0.13%)	5 (0.32%)	3 (0.05%)	0 (0.00%)	9 (0.22%)	0 (0.00%)	0 (0.00%)	4 (0.52%)	0 (0.00%)	0 (0.00%)	0 (0.00%)
Special sense, other disorders	0 (0.00%)	14 (0.38%)	3 (0.26%)	0 (0.00%)	0 (0.00%)	1 (0.06%)	16 (0.26%)	4 (0.64%)	21 (0.51%)	0 (0.00%)	2 (0.58%)	7 (0.91%)	0 (0.00%)	0 (0.00%)	5 (1.02%)
Psychiatric disorders	0 (0.00%)	932 (25.54%)	347 (29.63%)	263 (24.81%)	208 (26.40%)	317 (20.09%)	936 (15.15%)	127 (20.45%)	1077 (26.20%)	7 (30.43%)	46 (13.45%)	200 (25.97%)	78 (31.71%)	18 (24.00%)	101 (20.70%)
Gastro-intestinal system disorders	1 (100%)	828 (22.69%)	226 (19.30%)	295 (27.83%)	212 (26.90%)	593 (37.58%)	2438 (39.45%)	190 (30.60%)	1056 (25.69%)	9 (39.13%)	156 (45.61%)	166 (21.56%)	42 (17.07%)	19 (25.33%)	123 (25.20%)
Liver and biliary system disorders	0 (0.00%)	25 (0.69%)	8 (0.68%)	4 (0.38%)	8 (1.02%)	3 (0.19%)	22 (0.36%)	6 (0.97%)	25 (0.61%)	0 (0.00%)	2 (0.58%)	7 (0.91%)	6 (2.44%)	0 (0.00%)	2 (0.41%)
Metabolic and nutritional disorders	0 (0.00%)	170 (4.66%)	27 (2.31%)	45 (4.25%)	42 (5.33%)	46 (2.92%)	113 (1.83%)	25 (4.03%)	130 (3.16%)	0 (0.00%)	12 (3.51%)	14 (1.82%)	22 (8.94%)	3 (4.00%)	38 (7.79%)
Endocrine disorders	0 (0.00%)	3 (0.08%)	0 (0.00%)	0 (0.00%)	2 (0.25%)	1 (0.06%)	2 (0.03%)	1 (0.16%)	0 (0.00%)	0 (0.00%)	0 (0.00%)	0 (0.00%)	1 (0.41%)	0 (0.00%)	0 (0.00%)
Cardiovascular disorders, general	0 (0.00%)	40 (1.10%)	26 (2.22%)	29 (2.74%)	2 (0.25%)	28 (1.77%)	36 (0.58%)	25 (4.03%)	37 (0.90%)	0 (0.00%)	7 (2.05%)	25 (3.25%)	5 (2.03%)	0 (0.00%)	35 (7.17%)
Myo-, endo-, pericardial, and valve disorders	0 (0.00%)	2 (0.05%)	1 (0.09%)	0 (0.00%)	1 (0.13%)	1 (0.06%)	0 (0.00%)	1 (0.16%)	0 (0.00%)	0 (0.00%)	0 (0.00%)	0 (0.00%)	0 (0.00%)	1 (1.33%)	0 (0.00%)
Heart rate and rhythm disorders	0 (0.00%)	64 (1.75%)	44 (3.76%)	10 (0.94%)	11 (1.40%)	15 (0.95%)	88 (1.42%)	6 (0.97%)	60 (1.46%)	0 (0.00%)	3 (0.88%)	19 (2.47%)	2 (0.81%)	0 (0.00%)	2 (0.41%)
Vascular (extracardiac) disorders	0 (0.00%)	1 (0.03%)	0 (0.00%)	0 (0.00%)	0 (0.00%)	5 (0.32%)	5 (0.08%)	0 (0.00%)	2 (0.05%)	0 (0.00%)	1 (0.29%)	0 (0.00%)	0 (0.00%)	0 (0.00%)	0 (0.00%)
Respiratory system disorders	0 (0.00%)	45 (1.23%)	15 (1.28%)	5 (0.47%)	6 (0.76%)	16 (1.01%)	58 (0.94%)	5 (0.81%)	36 (0.88%)	0 (0.00%)	2 (0.58%)	9 (1.17%)	2 (0.81%)	0 (0.00%)	1 (0.20%)
Red blood cell disorders	0 (0.00%)	4 (0.11%)	2 (0.17%)	0 (0.00%)	0 (0.00%)	0 (0.00%)	1 (0.02%)	0 (0.00%)	3 (0.07%)	0 (0.00%)	0 (0.00%)	0 (0.00%)	0 (0.00%)	0 (0.00%)	0 (0.00%)
White cell and RES	0 (0.00%)	6 (0.16%)	0 (0.00%)	3 (0.28%)	2 (0.25%)	0 (0.00%)	11 (0.18%)	1 (0.16%)	15 (0.36%)	0 (0.00%)	0 (0.00%)	1 (0.13%)	0 (0.00%)	0 (0.00%)	1 (0.20%)
Platelet, bleeding, and clotting disorders	0 (0.00%)	15 (0.41%)	8 (0.68%)	2 (0.19%)	2 (0.25%)	5 (0.32%)	12 (0.19%)	0 (0.00%)	9 (0.22%)	0 (0.00%)	0 (0.00%)	1 (0.13%)	0 (0.00%)	0 (0.00%)	3 (0.61%)
Urinary system disorders	0 (0.00%)	44 (1.21%)	10 (0.85%)	30 (2.83%)	17 (2.16%)	13 (0.82%)	146 (2.36%)	8 (1.29%)	163 (3.96%)	1 (4.35%)	21 (6.14%)	5 (0.65%)	9 (3.66%)	1 (1.33%)	3 (0.61%)
Reproductive disorders (male)	0 (0.00%)	16 (0.44%)	3 (0.26%)	12 (1.13%)	1 (0.13%)	0 (0.00%)	6 (0.10%)	3 (0.48%)	3 (0.07%)	0 (0.00%)	1 (0.29%)	2 (0.26%)	0 (0.00%)	0 (0.00%)	0 (0.00%)
Reproductive disorders (female)	0 (0.00%)	12 (0.33%)	18 (1.54%)	5 (0.47%)	5 (0.63%)	7 (0.44%)	7 (0.11%)	2 (0.32%)	13 (0.32%)	0 (0.00%)	1 (0.29%)	0 (0.00%)	1 (0.41%)	1 (1.33%)	0 (0.00%)
Neoplasms	0 (0.00%)	0 (0.00%)	0 (0.00%)	0 (0.00%)	0 (0.00%)	0 (0.00%)	1 (0.02%)	0 (0.00%)	0 (0.00%)	0 (0.00%)	0 (0.00%)	0 (0.00%)	0 (0.00%)	0 (0.00%)	0 (0.00%)
Body-as-a-whole general disorders	0 (0.00%)	317 (8.69%)	81 (6.92%)	78 (7.36%)	56 (7.11%)	69 (4.37%)	422 (6.83%)	43 (6.92%)	306 (7.44%)	5 (21.74%)	15 (4.39%)	45 (5.84%)	16 (6.50%)	12 (16.00%)	44 (9.02%)
Application site disorders	0 (0.00%)	1 (0.03%)	0 (0.00%)	0 (0.00%)	0 (0.00%)	1 (0.06%)	0 (0.00%)	0 (0.00%)	0 (0.00%)	0 (0.00%)	00 (0.00%)	0 (0.00%)	0 (0.00%)	0 (0.00%)	0 (0.00%)
Resistance mechanism disorders	0 (0.00%)	0 (0.00%)	0 (0.00%)	0 (0.00%)	0 (0.00%)	0 (0.00%)	2 (0.03%)	0 (0.00%)	2 (0.05%)	0 (0.00%)	0 (0.00%)	0 (0.00%)	0 (0.00%)	0 (0.00%)	0 (0.00%)
Secondary terms—events	0 (0.00%)	36 (0.99%)	3 (0.26%)	4 (0.38%)	0 (0.00%)	19 (1.20%)	29 (0.47%)	7 (1.13%)	5 (0.12%)	0 (0.00%)	0 (0.00%)	1 (0.13%)	0 (0.00%)	0 (0.00%)	1 (0.20%)
Poison-specific terms	0 (0.00%)	0 (0.00%)	0 (0.00%)	0 (0.00%)	0 (0.00%)	0 (0.00%)	0 (0.00%)	1 (0.16%)	0 (0.00%)	0 (0.00%)	0 (0.00%)	0 (0.00%)	0 (0.00%)	0 (0.00%)	0 (0.00%)

Abbreviation: RES: reticuloendothelial system, SNRI: serotonin and norepinephrine reuptake inhibitor, SSRI: selective serotonin reuptake inhibitor, TCA: tricyclic antidepressants.

**Table 4:** Sensitivity analysis on the association between antidepressant medications and SAEs.

Antidepressants	Sensitivity	ROR (95% CI)	*p*-Value
SSRIs			
escitalopram	Age & Sex	0.96 (0.64–1.44)	0.85
	Age ≥ 60 years	1.34 (0.81–2.24)	0.259
	Causality	2.42 (1.24–4.73)	0.01
fluoxetine	Age and Sex	2.62 (1.58–4.36)	<0.001
paroxetine	Age and Sex	0.88 (0.45–1.72)	0.705
vortioxetine	Age and Sex	1.05 (0.55–1.99)	0.886
	Age ≥ 60 years	1.63 (0.71–3.76)	0.254
SNRIs			
duloxetine	Age and Sex	0.87 (0.62–1.22)	0.416
	Age ≥ 60 years	1.17 (0.76–1.81)	0.478
	Causality	0.60 (0.31–1.13)	0.11
venlafaxine	Age and Sex	1.32 (0.62–2.83)	0.476
TCA			
amitriptyline	Age and Sex	0.76 (0.52–1.13)	0.175
	Age ≥ 60 years	0.51 (0.28–0.92)	0.026
Others			
bupropion	Age and Sex	2.35 (1.26–4.37)	0.007
	Age ≥ 60 years	5.08 (2.17–11.89)	<0.001
	Causality	3.92 (1.52–10.13)	0.005

**Table 5:** Sensitivity analysis on association between SOC and SAE.

SOC	Sensitivity	ROR	*p*-Value
Skin and appendage disorders	Age and Sex	2.07 (1.29–3.31)	0.003
	Age ≥ 60 years	2.81 (1.52–5.21)	0.001
Central and peripheral nervous system disorders	Age and Sex	1.23 (0.87–1.74)	0.243
	Age ≥ 60 years	1.14 (0.70–1.87)	0.602
	Causality	3.08 (1.67–5.61)	<0.001
Psychiatric disorders	Age and Sex	0.42 (0.26–0.69)	<0.001
Gastro-intestinal system disorders	Age and Sex	0.30 (0.19–0.47)	<0.001
	Age ≥ 60 years	0.33 (0.18–0.62)	<0.001
	Causality	0.37 (0.17–0.83)	0.016
Liver and biliary system disorders	Age and Sex	10.66 (5.04–22.53)	<0.001
Metabolic and nutritional disorders	Age and Sex	4.12 (2.53–6.69)	<0.001
	Age ≥ 60 years	8.53 (4.93–14.75)	<0.001
	Causality	12.19 (5.47–27.20)	<0.001
Respiratory system disorders	Age and Sex	5.26 (2.54–10.92)	<0.001
	Age ≥ 60 years	8.76 (3.69–20.82)	<0.001
Body-as-a-whole general disorders	Age and Sex	1.82 (1.14–2.92)	0.012
	Age ≥ 60 years	2.00 (1.05–3.78)	0.034
	Causality	1.41 (0.50–3.98)	0.514

**Table 6:** Sensitivity analysis on the association of SOC-based ADEs with sex (men).

Male-Related SOC	Sensitivity	ROR	*p*-Value
Vision disorders	Age and Sex	0.05 (0.04–0.07)	<0.001
Special sense, other disorders	Age and Sex	0.47 (0.23–0.97)	0.04
Psychiatric disorders	Age and Sex	1.19 (1.10–1.30)	<0.001
	Age ≥ 60 years	1.14 (1.01–1.28)	<0.001
	Causality	0.82 (0.70–0.97)	0.021
Gastro-intestinal system disorders	Age and Sex	0.75 (0.70–0.81)	<0.001
	Age ≥ 60 years	0.74 (0.66–0.82)	<0.001
	Causality	1.42 (1.22–1.65)	<0.001
Urinary system disorders	Age and Sex	1.78 (1.44–2.21)	<0.001
	Age ≥ 60 years	2.15 (1.66–2.80)	<0.001
	Causality	0.49 (0.31–0.76)	0.002
Cardiovascular disorders, general	Age and Sex	1.32 (1.02–1.70)	0.032
Heart rate and rhythm disorders	Age and Sex	0.66 (0.48–0.92)	0.013
	Causality	2.93 (1.45–5.93)	0.003
Body-as-a-whole general disorders	Age and Sex	0.87 (0.75–1.00)	0.044
	Age ≥ 60 years	0.80 (0.65–0.97)	0.026
Sin and appendage disorders	Age ≥ 60 years	1.24 (1.01–1.53)	<0.001
Central and peripheral nervous system	Causality	0.85 (0.72–1.00)	0.046
Metabolic and nutritional disorders	Age ≥ 60 years	8.53 (4.93–14.75)	<0.001
Liver and biliary system disorders	Causality	0.20 (0.08–0.52)	0.001
Platelet, bleeding, and clotting disorders	Age ≥ 60 years	2.91 (1.27–6.65)	0.011

**Table 7:** Sensitivity analyses on predictors of antidepressant-associated SAE risk.

Predictors	Sensitivity	OR	*p*-Value
Fluoxetine use	Age and Sex	3.13 (1.88–5.22)	<0.001
Antiparkinsonian treatment	Age and Sex	8.86 (3.76–20.86)	<0.001
	Age ≥ 60 years	5.67 (2.22–14.50)	<0.001
	Causality	46.97 (10.81–204.14)	<0.001
Antidementia treatment	Age and Sex	2.95 (1.38–6.33)	0.005
	Age ≥ 60 years	1.56 (1.19–2.04)	0.001
No. of concomitant Meds	Age and Sex	0.87 (0.79–0.96)	0.004
	Causality	1.12 (1.01–1.25)	0.036
Aging	Age ≥ 60 years	11.16 (4.28–29.09)	<0.001

## Data Availability

The data presented in this study are available on request from the corresponding author and KIDS, due to the inclusion of patient information and ethical concerns.

## Abbreviations

The following abbreviations are used in this manuscript:

ADE	Adverse Drug Events
KIDS KAERS DB	Korean Adverse Drug Reporting System Database
MDD	Major Depressive Disorder
OR	Odds Ratio
PD	Pharmacodynamics
PK	Pharmacokinetics
ROR	Reporting Odds Ratio
RWD	Real World Data
SAE	Serious Adverse Events
SNRI	Serotonin Norepinephrine Reuptake Inhibitors
SOC	System Organ Class
SSRI	Selective Serotonin Reuptake Inhibitor
TCA	Tricyclic Antidepressants
WHO	World Health Organization
